# *In silico* Characterization of the Heme Oxygenase 1 From Bottlenose Dolphin (*Tursiops truncatus*): Evidence of Changes in the Active Site and Purifying Selection

**DOI:** 10.3389/fphys.2021.711645

**Published:** 2021-08-12

**Authors:** Carlos A. Reyes-Ramos, Ramón Gaxiola-Robles, José Pablo Vázquez-Medina, Luis Javier Ramírez-Jirano, Oscar Kurt Bitzer-Quintero, Tania Zenteno-Savín

**Affiliations:** ^1^Centro de Investigaciones Biológicas del Noroeste, S.C. Planeación Ambiental y Conservación, La Paz, Mexico; ^2^Hospital General de Zona No. 1, Instituto Mexicano del Seguro Social, La Paz, Mexico; ^3^Department of Integrative Biology, University of California, Berkeley, Berkeley, CA, United States; ^4^Centro de Investigación Biomédica de Occidente, Instituto Mexicano del Seguro Social, Guadalajara, Mexico

**Keywords:** anti-inflammatory response, dolphin, heme oxygenase, immunology, inflammation

## Abstract

Cetacea is a clade well-adapted to the aquatic lifestyle, with diverse adaptations and physiological responses, as well as a robust antioxidant defense system. Serious injuries caused by boats and fishing nets are common in bottlenose dolphins (*Tursiops truncatus*); however, these animals do not show signs of serious infections. Evidence suggests an adaptive response to tissue damage and associated infections in cetaceans. Heme oxygenase (HO) is a cytoprotective protein that participates in the anti-inflammatory response. HO catalyzes the first step in the oxidative degradation of the heme group. Various stimuli, including inflammatory mediators, regulate the inducible HO-1 isoform. This study aims to characterize HO-1 of the bottlenose dolphin *in silico* and compare its structure to the terrestrial mammal protein. Upstream HO-1 sequence of the bottlenose dolphin was obtained from NCBI and Ensemble databases, and the gene structure was determined using bioinformatics tools. Five exons and four introns were identified, and proximal regulatory elements were detected in the upstream region. The presence of 10 α-helices, three 3_10_ helices, the heme group lodged between the proximal and distal helices, and a histidine-25 in the proximal helix serving as a ligand to the heme group were inferred for *T. truncatus*. Amino acid sequence alignment suggests HO-1 is a conserved protein. The HO-1 “fingerprint” and histidine-25 appear to be fully conserved among all species analyzed. Evidence of positive selection within an α-helix configuration without changes in protein configuration and evidence of purifying selection were found, indicating evolutionary conservation of the coding sequence structure.

## Introduction

Many reports suggest that mammals returned to the water in separate lineages and times. Therefore, the three marine orders (Cetacea, Sirenia, and Carnivora) have separate evolutionary origins (Irwin and Árnason, 1994; Uhen, 2007). Cetaceans returned to the ocean approximately 53–56 million years ago (Thewissen et al., 2007). This transition from a terrestrial to an aquatic lifestyle poses different challenges, including but not limited to the change of pathogens and their pathogen-response proteins and the oxygen source (since they must dive to obtain prey or any food source). Evidence of selective pressures can be found in distinct cetacean genes like the toll-like receptor (TLR) 4 (a recognition receptor of pathogen-associated molecular patterns which mediates the innate immune system), hemoglobin, (alpha and beta) myoglobin and endothelin (Tian et al., 2016), suggesting an adaptive evolution to the aquatic lifestyle (Shen et al., 2012). Moreover, antioxidants and other proteins (e.g., glutathione peroxidase 2 and haptoglobin) showed amino-acid changes in cetaceans (Yim et al., 2014).

Marine mammals, compared to terrestrial mammals, have an increased blood volume, hematocrit, and hemoglobin concentration, as well as muscle myoglobin concentration (Lenfant et al., 1970; Kooyman and Ponganis, 1998; Dolar et al., 1999; Castellini et al., 2006), which allows them to store and conserve oxygen efficiently. Also, physiological responses like bradycardia, vasoconstriction, pressure, and hypoxemic tolerance are part of marine mammals’ diving capacities (Ponganis, 2019). Even though the fluctuations in blood flow and oxygen saturation can cause injuries (e.g., reperfusion injuries driven by ischemic inflammation), marine mammals seem to tolerate these conditions without the associated injuries reported in terrestrial mammals (Allen and Vázquez-Medina, 2019). Similarly, there is evidence that diving mammals have a robust antioxidant defense system (Vázquez-Medina et al., 2007, 2012; Zenteno-Savín et al., 2002, 2012) as compared to non-diving mammals. The oxidative stress caused by diving may generate a protective preconditioning effect by modulating antioxidant gene transcription (Zhang et al., 2010; Wang et al., 2018). It has been speculated that it is also an anti-inflammatory strategy.

Heme oxygenase (HO) is a cytoprotective protein that participates in the anti-inflammatory response. HO catalyzes the first step of oxidative degradation of the heme group, a pro-oxidant molecule that can destabilize nucleic acids, proteins, and lipids (Choi and Alam, 1996; Morse and Choi, 2002), as well as the liberation of biliverdin, which transformed into bilirubin is an antioxidant and anti-inflammatory pigment (Stocker, 2004; Kapitulnik and Maines, 2009). The inducible HO-1 isoform is regulated by various stimuli, including inflammatory mediators, factors associated with oxidative stress (Terry et al., 1998; Maines and Panahian, 2001), ultraviolet radiation, hyperthermia, ischemia-reperfusion (I/R), heavy metals, hydrogen peroxide, and endotoxins (Maines, 1992; Choi and Alam, 1996; Abraham et al., 2002). HO is mainly upregulated by the activation of the oxidant-responsive transcription factor, nuclear factor (erythroid-derived 2)-like 2 (Nrf2) (Kapturczak et al., 2004; Paine et al., 2010).

Two genetically distinct and functional isoforms of HO have been characterized, the inducible isoform (HO-1) and the constitutive isoform (HO-2) (Trakshel et al., 1986). A third isoform (HO-3), similar to HO-2 (90%) in its amino acid sequence, has been characterized, but it has low enzymatic activity (McCoubrey et al., 1997). Subcellular localization of HO-1 has been primarily reported in the endoplasmic reticulum (ER) (Gottlieb et al., 2012) [with a C-terminus transmembranal region that aids anchoring to the ER (Kikuchi et al., 2005)], but also in plasma membrane caveolae (Kim et al., 2004), mitochondria (Slebos et al., 2007), and nucleus (Biswas et al., 2014). In hypoxic conditions, a C-terminal truncated protein (28 kDa) was found in the nucleus (Lin et al., 2007). HO-1 is a 32–33 kDa protein (Keyse and Tyrrell, 1989) mostly composed of α-helix, with two helices (distal and proximal) surrounding the heme pocket, as suggested by crystallography (Kikuchi et al., 2005). Also, HO-1 has conserved regions such as the HO “fingerprint,” which consists of 26 residues located in the distal helix (residues 125–150 in human HO-1) (Maines, 1992). In the proximal helix, the presence of a highly conserved histidine (His-25), identified as a heme-ligand, has been reported; if it is replaced by an alanine, inactivation of the protein ensues (Wilks et al., 1995).

The regioselectivity of the HO reaction is controlled mostly sterically, determined by the interaction of the basic residues near the propionates of the heme group (Kikuchi et al., 2005). The mutation of Arg-183 for Glu-183 in rat HO-1, leads to the formation of δ-isomers of biliverdin IX, caused by the rotation of the heme group (Zhou et al., 2000). In *Pseudomonas aeruginosa* HO, in which rotation of the heme group is nearly 100°, a mixture of β- and δ-isomers of biliverdin IX is produced (Ratliff et al., 2001; Friedman et al., 2004). Likewise, a substitution of Lys-132 with Ala in *P. aeruginosa* HO induces the production of α-biliverdin (Fujii et al., 2004). Altogether, these results indicate the steric control of the HO reaction.

The cytoprotective function of HO is also due to the liberation of carbon monoxide [CO (described below)] and iron, which can be synthesized as ferritin, a cytoprotectant protein (Vile et al., 1994). Carbon monoxide is a relatively stable diatomic molecule in biological systems without unpaired electrons, binding to hemeproteins and metalloenzymes (Coburn and Forman, 1987; Maines, 1997). Although CO can be considered toxic because it has more affinity for hemoglobin than oxygen, thus decreasing oxygen transport to tissues (Hall et al., 2007), it is endogenously generated in low concentrations. It has a role in tissue protection (Kevin and Laffey, 2008). As a product of heme degradation, CO can suppress proinflammatory cytokine production and decrease the mortality rate in septic mice (Otterbein et al., 2000; Morse et al., 2003).

Several signal transduction pathways are modulated by CO, including guanylyl cyclase, cyclic GMP (Morita et al., 1995), and p38 mitogen-activated protein kinase (MAPK) (Otterbein et al., 2000), contributing to regulate the expression of vasoconstrictor, proinflammatory, and procoagulant molecules. Presumably, this range of action accounts for CO’s ability to promote vasodilation, inhibit inflammation (Otterbein et al., 2000), and suppress apoptosis (Petrache et al., 2000). It has been hypothesized that high concentrations of hemoproteins are the source of high levels of CO in the blood in deep-diving marine mammals, such as the northern elephant seal (*Mirounga angustirostris*), and that CO may reduce or prevent tissue damage caused by chronic hypoxemia and I/R events (Tift and Ponganis, 2019).

Toll-like receptors recognize viral nucleic acid and bacterial components such as lipopolysaccharides (LPS) and lipoteichoic acid (Akira et al., 2006). Following stimulation with LPS, TLR4 can activate myeloid differentiation primary response 88 (MyD88) (Jia et al., 2014), which triggers the liberation of proinflammatory cytokines and stimulates the translocation of nuclear factor kappa B (NFκB) (Calippe et al., 2008). This transcription factor regulates the expression of genes involved in the immune, inflammatory (Siebenlist et al., 1994; Barnes and Karin, 1997), and antiviral responses (Boehm et al., 1997). In the whole blood of Weddell seals (*Leptonychotes weddellii*) exposed to LPS, the cytokine response was lower compared to human blood under the same conditions; this may be explained by a serum-derived factor (Bagchi et al., 2018).

It is common to see severe injuries in marine mammals, such as dolphins and humpback whales (*Megaptera novaeangliae*), caused by boats and fishing nets, but these animals do not show signs of serious infections (Angliss and DeMaster, 1998; Andersen et al., 2008). Also, the healing of skin biopsies and the absence of infection in open wounds in bottlenose dolphins (*Tursiops truncatus*) (Bruce-Allen and Geraci, 1985; Zasloff, 2011) suggest that there is an adaptive response to tissue damage and associated infections in cetaceans.

Although HO and its anti-inflammatory activity have been characterized in terrestrial mammals, they are poorly understood in marine mammals, including cetaceans. The objectives of this study were to characterize the anti-inflammatory enzyme HO-1 from bottlenose dolphin *in silico* and to compare its structure to that of HO-1 from terrestrial mammals. Potential changes in the inflammatory resolution response associated with structural changes in HO-1 in dolphins due to selective pressure resulting from the adaptation to a marine lifestyle, including exposure to marine pathogens and diving, are discussed.

## Materials and Methods

### Motif-Based Sequence Analysis of Heme Oxygenase-1 Gene Upstream

Upstream HO-1 sequence of diverse eukaryotes were obtained from NCBI,^1^ and Ensemble database for intron/exon boundaries of *hmox1* of bottlenose dolphin (Access number ENSTTRG00000007342.1). Multiple Expression motifs for Motif Elicitation (MEME) suite 5.1.1 (Bailey and Elkan, 1995) were used for searching motifs represented as position-dependent letter-probability matrices. Additionally, regulatory elements of the human HO-1 gene previously identified (Lavrovsky et al., 1994) were searched in JASPAR and analyzed with Motif Alignment and Search Tool (MAST) (Bailey and Gribskov, 1998) to determine binding sites of transcriptional factors in the upstream region of HO-1 gene. To determine the best match-score, MAST determines the best match in the sequence to each motif, and these are combined into a score for the total match between the complete motif set and the sequence, which results in an *E*-value for each sequence. Also, a position *p*-value is given, which is defined as the probability of a same length random subsequence containing some match with a similar or better score. A motif occurrence is only given when the *p*-value is less than 0.0001. Furthermore, to verify if these motifs harbor binding for the transcriptional factors identified in this study, a search for candidate cis-regulatory elements (cCREs) of HO-1 was conducted from the ENCODE portal^2^ in the human genome assembly GRCh38. This search shows the cCREs are located between the first transcription start site of HMOX1 and up to 10 kb upstream.

### Bottlenose Dolphin Heme Oxygenase-1 Secondary and Tertiary Structure

To analyze the secondary and tertiary structure of *T. truncatus* HO-1, a sequence was obtained from the NCBI database (see text footnote 1) (GenBank accession number XP_004315933.1). The prediction of secondary structures was performed with the STRIDE interphase (Heinig and Frishman, 2004); the template search and the modeling of the three-dimensional structure of the protein was performed using Phyre2 at the website^3^ (Kelley et al., 2015). PyMol^4^ was used for modeling interactions between substrates/products and the active site. For modeling purposes, the crystal structure of *H. sapiens* HO-1 (PDB 1N45; Schuller et al., 1999) in complex with the heme group was used, and the level of alignment was calculated with the root mean square deviation (RMSD) to numerically evaluate the visual differences of their 3D structures. Additionally, tertiary structure alignment of HO-1 of vertebrates with different physiological adaptations (hypoxia-sensitive, hypoxia-tolerant, high-altitude, cold adapted, diving capacity) was predicted. The selected species were rat (*Rattus rattus*), mummichog (*Fundulus heteroclitus*), naked-mole rat (*Heterocephalus glaber*), sperm whale (*Physeter catodon*), bottlenose dolphin (*T. truncatus*), California sea lion (*Zalophus californianus*), deer mouse (*Peromyscus maniculatus bairdii*) and common frog (*Rana temporaria*). All structures were aligned with the human HO-1. The level of alignment is indicated by RMSD, being <0.3 very good alignment, 0.3–0.8 moderate alignment, 0.8–1.0 poor alignment, and >1.0 very poor alignment.

### Heme Oxygenase-1 Amino Acid Sequence Alignment

The HO-1 amino-acid sequence analyses of several eukaryotes was performed with the ClustalW2 algorithm (Thompson et al., 1994), positions with <95% site coverage were eliminated, and a heatmap was constructed. Also, a multiple alignment of cetaceans and primates were done with CLUSTAL Omega.^5^ Species used for the alignment included bottlenose dolphin (*T. truncatus)*, minke whale (*Balaenoptera acutorostrata scammoni*), killer whale (*Orcinus orca*), narrow-ridged finless porpoise (*Neophocaena asiaeorientalis asiaeorientalis*), beluga (*Delphinapterus leucas*), sperm whale (*Physeter catodon*), human (*H. sapiens)*, gorilla (*Gorilla gorilla gorilla*), bonobo (*Pan paniscus*), silvery gibbon (*Hylobates moloch*), Ugandan red colobus (*Piliocolobus tephrosceles*), baboon (*Papio anubis*), and crab-eating macaque (*Macaca fascicularis*).

### Phylogenetic Analysis of Heme Oxygenase 1 in Mammals

The HO-1 amino acid sequences from distinct mammalian orders were obtained from the NCBI database (Table 1), and multiple alignments were performed with the ClustalW2 algorithm (Thompson et al., 1994). A phylogenetic tree was constructed using MEGA X (Kumar et al., 2018) with the maximum likelihood (ML) method and the Jones–Taylor–Thornton (JTT) substitution model, with 1,000 bootstrap replicates. A discrete Gamma distribution was used to model evolutionary rate differences among sites [5 categories (+ G, parameter = 0.6641)], and positions with less than 95% site coverage were eliminated, leaving 288 positions in the final data set. The tree was rooted to the HO-1 sequence from the koala (*Phascolarctos cinereus*).

**TABLE 1:** Heme oxygenase-1 sequences used for amino acid alignment and phylogenetic analysis with their GenBank accession number.

Scientific name	Accession number GenBank
*Homo sapiens*	NP_002124.1
*Tursiops truncatus*	XP_004315933.1
*Mus musculus*	NP_034572.1
*Balaenoptera acutorostrata scammoni*	XP_007165768.1
*Zalophus californianus*	XP_027451406.1
*Ursus arctos horribilis*	XP_026355496.1
*Trichechus manatus latirostris*	XP_004373867.1
*Loxodonta africana*	XP_023415740.1
*Neophocaena asiaeorientalis asiaeorientalis*	XP_024599272.1
*Odobenus rosmarus divergens*	XP_004416157.2
*Delphinapterus leucas*	XP_022455673.1
*Physeter catodon*	XP_007101964.2
*Vulpes vulpes*	XP_025866739.1
*Macaca fascicularis*	NP_001271612.1
*Papio anubis*	XP_021777178.1
*Hylobates moloch*	XP_032003242.1
*Piliocolobus tephrosceles*	XP_023077866.1
*Gorilla gorilla gorilla*	XP_030861390.1
*Pan paniscus*	XP_003821555.1
*Orcinus orca*	XP_004286250.1
*Callorhinus ursinus*	XP_025737813.1
*Phoca vitulina*	XP_032272596.1
*Phascolarctos cinereus*	XP_020838126.1

The divergence time between the selected groups of mammals was estimated by the RelTime-ML algorithm implemented in MEGA X (Tamura et al., 2012). Briefly, time estimates are computed based on branch lengths optimized by ML, which is a robust statistical method. The ML topology analysis was used as the starting tree, with the outgroup taxon defined manually. The GTR + I + G model was implemented with five discrete gamma categories. The divergence time (mostly from fossil records) of the bottlenose dolphin with the killer whale [8.1–11.7 million years ago (Mya)], minke whale (31.1–35 Mya), and human (91–101 Mya) was used to calibrate the relative node ages from TimeTree^6^ (Kumar et al., 2017).

### Detection of Positive/Negative Selection

To determine selective pressures and quantify the impact of natural selection on the molecular evolution of cetacean and primate HO-1, non-synonymous/synonymous substitution rates (ω = dN/dS) were calculated using SWAAP 1.0.2^7^ using a sliding window method (window size = 90, step size = 9). If ω = 1, amino acid substitutions may be largely neutral; ω > 1 is evidence of positive selection, and ω < 1 is consistent with negative (purifying) selection. An improved statistical method was used in the Datamonkey webserver (Pond and Frost, 2005), which computed non-synonymous and synonymous substitutions at each codon position. An ML method with default settings applied in this web, fixed effects likelihood (FEL), which directly estimates dN and dS based on a codon-substitution model, was used. Also, the 3D model protein and STRIDE interphase were used to evaluate the functional significance of selected sites located within or near the predicted 3D structure’s functional domain.

## Results

### Gene Characterization of Bottlenose Dolphin *hmox1*

**FIGURE 1:**
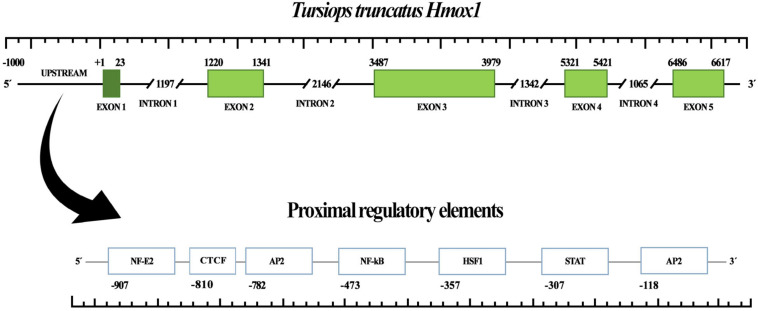
Heme oxygenase 1 gene (*hmox1*) structure of bottlenose dolphin (*Tursiops truncatus*). Upstream region, exons, and introns are shown. Exons/introns boundaries were obtained from Ensembl. Proximal regulatory elements are shown.

**FIGURE 2:**
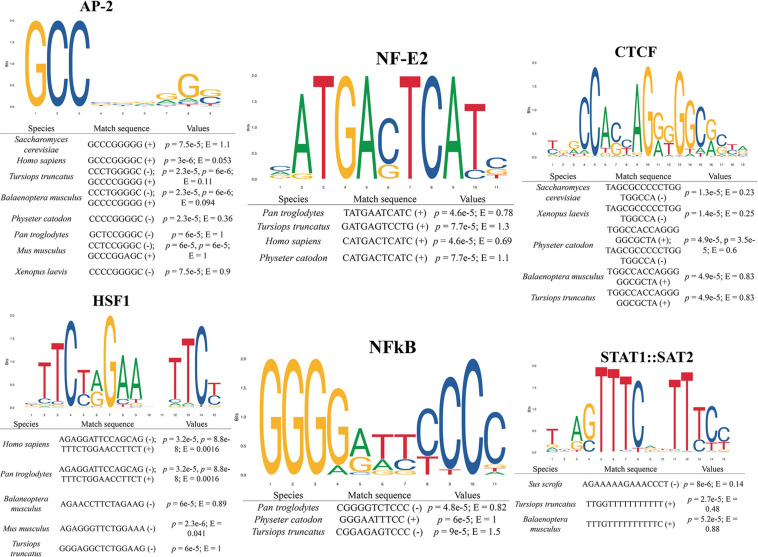
Binding site prediction for transcription factors in the promoter region of distinct taxa heme oxygenase 1 gene (*hmox1*). *E*-values and prediction *p*-values are shown. (+) and (–) indicate forward and reverse strand, respectively.

Gene structure of bottlenose dolphin *hmox1* was determined using bioinformatic tools (Figure 1). The open reading frame is 864 bp; five exons and four introns were identified. Proximal regulatory elements were detected in the upstream region (up to 1,000 bp) (Figure 1), including the following transcriptional factors, activating protein 2 (AP-2), nuclear factor kappa-light-chain-enhancer of activated B cells (NF-κB), heat shock factor 1 (HSF1), signal transducers, and transcription activators (STAT1:STAT2), as well as nuclear factor erythroid 2 (NF-E2) and CCCTC-binding factor (CTCF), a multifunctional transcription factor. Moreover, binding sites for those transcription factors were detected in the upstream region of *hmox1* (Figure 2). The best match-score for AP-2 and NF-E2 binding sites with *E*-value of 0.053 and 0.69, respectively, was calculated for humans; the best match for NF-kB, with *E* = 0.82, was for the chimpanzee; for HSF1, the best match with *E* = 0.0016 was found in the two primates sequences; for STAT1:STAT2, the best match with *E* = 0.14 was obtained for the pig, and for CTCF, the best match with *E* = 0.23 was calculated for yeast. For all matches in all species selected in this study, the *E*-values were less than 1.5 and all the position *p*-values were less than 0.0001. When searching for cCREs, only the transcription factors with ChIP-seq experiments for the given transcription factor with peaks intersecting the selected cis-regulatory element (CRE) were used to verify the binding sites of the transcriptional factors identified by MEME (Table 2).

**TABLE 2:** cis-Regulatory elements of human heme oxygenase 1 (HO-1) with ChIP-seq experiments.

Factor	# of experiments that support transcriptional factor binding	Genome assembly/References
CTCF	70	GRCh38
NF-E2	1	GRCh38
HSF-1	1	GRCh38
STAT1	2	GRCh38
STAT2	1	GRCh38
NF-κB	–	Lavrovsky et al., 1994
AP-2	–	Lavrovsky et al., 1994

*CTCF, CCCTC-binding factor; NF-E2, nuclear factor erythroid 2; HSF-1, heat shock factor 1; STAT1, STAT2, signal transducers and transcription activators; NF-κB, nuclear factor kappa-light-chain-enhancer of activated B cells; AP-2, activating protein 2.*

### *Tursiops truncatus* HO-1 Protein Modeling

The three-dimensional bottlenose dolphin HO-1 was obtained by modeling from the crystallized structure of HO-1 fused with cytochrome P450 reductase from the rat (*Rattus norvegicus*) (Protein Data Bank no. 6J7A) with 80% identity between both HO-1. The composition of secondary structures using the STRIDE web platform revealed ten α-helices. An α-helix at the C-terminal end belongs to the transmembrane domain of anchorage to the endoplasmic reticulum; three 3_10_ helices were inferred for *T. truncatus* HO-1 (Supplementary Figure 1). The heme group is lodged between two helices, the proximal and distal. To model the active site, alignment was set to human HO-1 in complex with the heme group (Supplementary Figure 2). Histidine-25 (His-25) is found in the proximal helix, which serves as a ligand to the heme group (2.1 Å). The heme group has two propionates that orient the heme group and position the α-meso carbon for hydroxylation. Two of the amino-acids that interact with the heme group’s propionate have distances to the propionate group of 4.4 and 3 Å for lysine (Lys)-179, and 6 and 4.4 Å for arginine (Arg)-183 (bottlenose dolphin and human, respectively). Interestingly, Lys-18 of bottlenose dolphin was found interacting with both propionates at a distance as near as 1.4 Å, which could indicate an electrostatic interaction, while the distance to Lys-18 of human is 4.2 Å. Altogether, these three residues are found proximal to these propionates and their interactions determine the direction of the heme group.

### Conformational Differences and Substitutions of the Heme Oxygenase-1 Structure-Prediction

**FIGURE 3:**
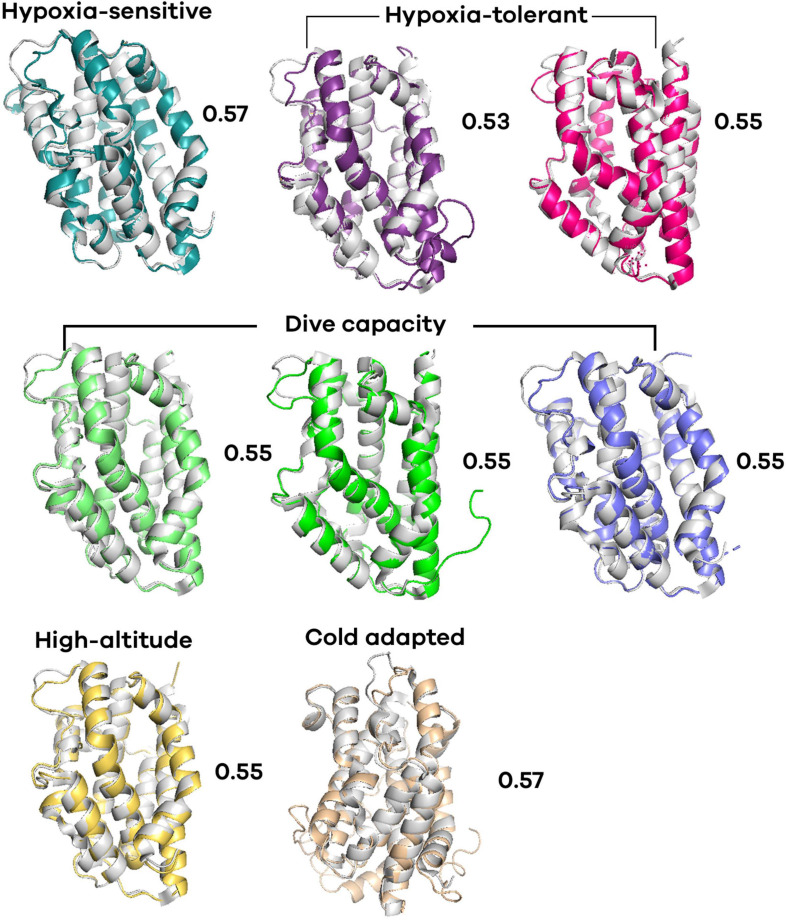
Predicted tertiary structure alignment of heme oxygenase 1 (HO-1) of vertebrates with different physiology adaptations. All structures were aligned with the human HO-1. Rat (*Rattus rattus*) (cyan), mummichog (*Fundulus heteroclitus*) (purple), naked-mole rat (*Heterocephalus glaber*) (magenta), sperm whale (*Physeter catodon*) (opaque green), bottlenose dolphin (*Tursiops truncatus*) (green), California sea lion (*Zalophus californianus*) (blue), deer mouse (*Peromyscus maniculatus bairdii*) (yellow), common frog (*Rana temporaria*) (wheat). The root mean square deviation (RMSD) values are indicated.

**FIGURE 4:**
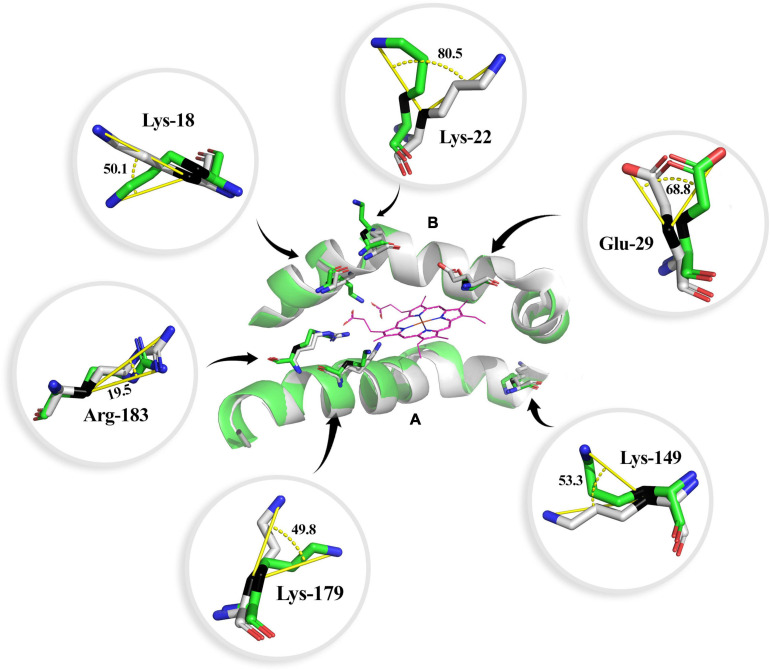
Heme group interaction site of the bottlenose dolphin heme oxygenase 1 (HO-1) alignment to human HO-1. Angle of rotation is shown in degrees. The carbon β is indicated in black. The distal **(A)** and proximal **(B)** helix are shown. Carbon atoms are shown in green for bottlenose dolphin and gray for human (except for the heme group, in which carbon atoms are shown in purple), nitrogen atoms in blue, and oxygen in red.

When comparing distinct HO-1 tertiary structures of animals with different physiological adaptations against the HO-1 of human, moderate level of alignment (RMSD ∼ 0.5) was found (Figure 3). The protein alignment revealed several substitutions between bottlenose dolphin and human HO-1 (Supplementary Figure 3). Numerous sites and regions that are part of the HO-1 configuration and regulation have been identified. Based on this, we analyzed these arrangements in the hypoxia-tolerant naked-mole rat (*H. glaber*) and the mumichog (*F. heteroclitus*), the hypoxia-sensitive rat (*R. norvegicus*), the high-altitude adapted deer mice (*P. maniculatus bairdii*), the cold adapted common frog (*R. temporaria*), and marine mammals with different diving capacities, the California sea lion (*Z. californianus*), sperm whale (*P. catodon*) and bottlenose dolphin (*T. truncatus*) HO-1, and compared them with the human (*H. sapiens*) HO-1 (Table 3 and Supplementary Figure 4). In the HO-1 “fingerprint” region, four amino-acids were found with a plane of rotation different than that in humans (Table 3); remarkably, six amino-acids that interact with the heme group have a rotation on the carbon β (Cβ) (Figure 4). Furthermore, Arg-183 has its guanidine side chain inverted 93.8°. All the predicted tertiary structures analyzed in this study have rotated amino-acids when compared to the human HO-1. However, *T. truncatus* HO-1 was the one with more remarkable changes.

**TABLE 3:** Residues of bottlenose dolphin heme oxygenase 1 (HO-1) with different rotation than that in human HO-1.

Residue	Rotation angle (°)	Location/function
Lys-18*	50.1	Interaction with the heme propionates
Lys-22*	80.5	Near the heme propionates
Glu-29*	68.8	Heme pocket contact residue
Lys-149*	53.3	Part of the HO signature
Lys-179*	49.8	Interaction with the heme propionates
Arg-183*	19.5	Interaction with the heme propionates
Lys-153	52.3	Located within the HO-1 fingerprint
Val-130	124.2	Located within the HO-1 fingerprint
Gln-145	112.9	Located within the HO-1 fingerprint
Val-146	48.5	Located within the HO-1 fingerprint

*Residues with major interaction in the heme pocket; shown in further detail in Figure 4. Lys, Lysine; Glu, glutamic acid; Arg, arginine; Val, valine; Gln, glutamine.

### Amino Acid Sequence Alignment of Eukaryotic HO-1

**FIGURE 5:**
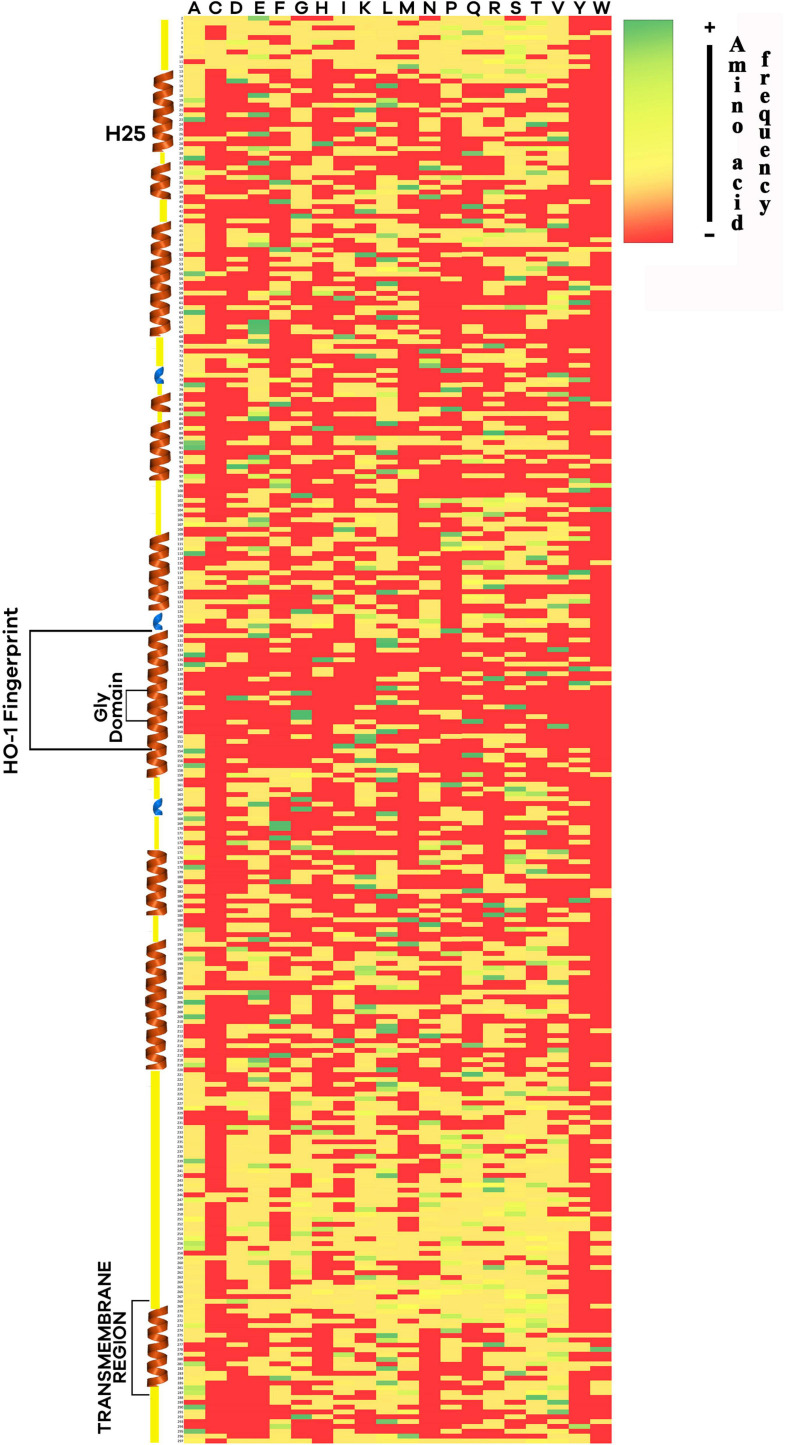
Heat map constructed with the amino-acid sequence of heme oxygenase 1 (HO-1) from 245 eukaryotic species. Tertiary structures predicted from mammal sequences such as alpha-helix (brown) and 3_10_ helix (blue) are shown. Histidine-25, HO-1 fingerprint, the glycine domain, and the transmembrane region are indicated. Color indicates the frequency of an amino-acid across all the sequences, green for more frequency, red for none.

A heat map of the HO-1 amino-acid sequence of 245 eukaryotic species was constructed in order to see if there are areas of conservation across Eukarya (Figure 5). The HO-1 “fingerprint” has been reported in most of the proteins from the species studied so far, suggesting that this is an important site for the protein’s catalytic function. Within this region, a glycine domain (Gly-Asp-Leu-Ser-Gly-Gly), located on the heme group, was found; this region can provide the flexibility required for the binding of the substrate (heme) and the release of the product (biliverdin) (Wilks, 2002). Interestingly, only three groups (plants, flies, and fungi) analyzed in this study were found to lack the glycine domain. His-25, identified as the residue closest to the iron within the heme group (2.1 Å), is almost fully conserved among all species analyzed; a substitution of this residue causes inactivation of the enzyme, although its activity can be restored with exogenous imidazole (Wilks et al., 1995). At the C-terminus, a transmembrane region that allows for the protein to anchor to the endoplasmic reticulum was found. The deduced amino acid sequences of mammalian HO-1 are shown in Table 1. A percentage of identity > 70% was observed between species, except for the koala (*P. cinereus*), which had a percentage between 60–70% compared to placental mammals. The highest levels of identity were observed among individual orders, exceeding 90%. This suggests that HO-1 is a conserved protein in mammals and highly conserved regions were found in the HO-1 selected for alignment (Supplementary Figure 3). Additionally, cysteine, the least abundant residue, was found in almost all the eukaryotic species included in this study with the exception of primates and marine mammals.

### Selective Pressure of *hmox1* in Cetaceans and Primates

**FIGURE 6:**
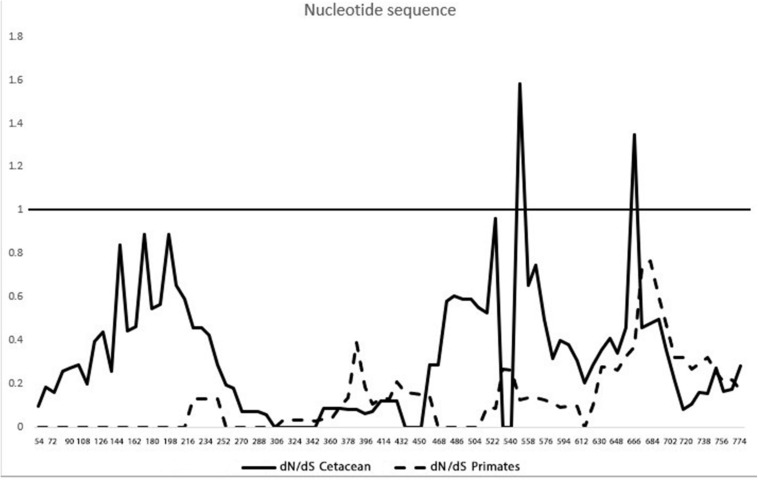
Different non-synonymous/synonymous substitution (dN/dS) rates along the coding sequence of the heme oxygenase-1 gene (*hmox1*). The solid line is the pairwise dN/dS rate within cetaceans. The dashed line represents the pairwise dN/dS rate in primates.

The dN/dS ratio was calculated by the sliding window method (Figure 6) for cetaceans and primates. Only two sites under positive selection were detected among cetaceans and none among primates, whereas most of the sites appeared to be under purifying selection for both groups. To identify the sites under selective pressure, an ML method, FEL, was used. Two sites (50 and 196, *p* = 0.032; *p* = 0.009, respectively) under positive selection and five sites under purifying selection (more detail provided in Supplementary Figure 3) were detected with this method.

### Phylogenetic Analysis of Mammalian Heme Oxygenase 1

**FIGURE 7:**
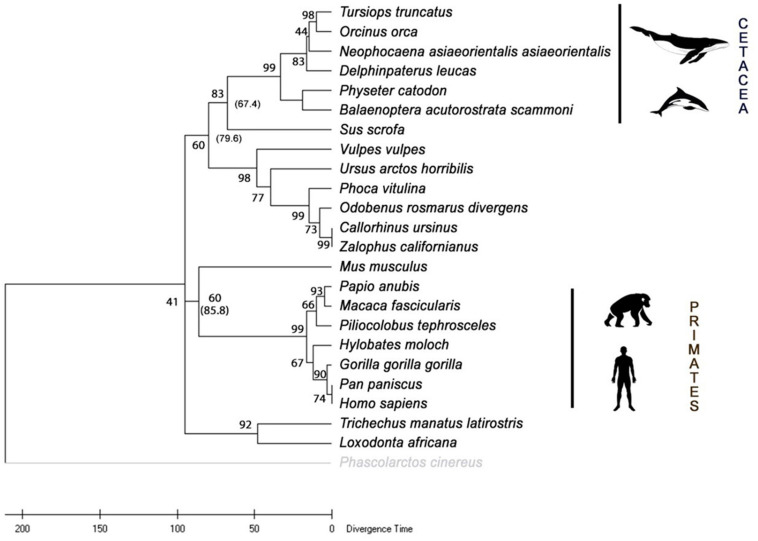
Consensus phylogenetic tree with divergence time of various mammalian heme oxygenase-1 (HO-1). The analysis included 24 amino acid sequences. All positions with less than 95% site coverage were eliminated. There was a total of 288 positions in the final data set. The tree was rooted to the HO-1 sequence from the koala (*Phascolarctos cinereus*). Bootstrap values are indicated. Divergence time in millions of years (Mya) indicated in parenthesis.

The phylogenetic tree (Figure 7) was constructed from 24 HO-1 sequences (Table 1) using the maximum likelihood method. The HO-1 of marine mammals was grouped according to their order (Cetacea, Sirenia, and Carnivora) and their closest terrestrial relatives. The phylogenetic tree was rooted to the HO-1 of the koala, a marsupial. The order Primates was grouped with a 99% bootstrap value. The cetaceans were grouped with a bootstrap value of 99%, and this group with the domestic pig (representative of the terrestrial artiodactyls), with a bootstrap value of 83%. The manatee was grouped with the African elephant (superorder Afrotheria) with a value of 92% and the carnivores with a value of 98%. These results support the suggestion that marine mammals are an artificial group with more significant similarity to their terrestrial relatives than with each other.

The time tree of the selected mammalian species was estimated with RelTime from DNA sequence data of *hmox1* based on the phylogenetic tree constructed in this study with ML topology (Figure 7). For the artiodactyls, the estimated date of divergence was 67.4 Mya, for the carnivores 79.6 Mya, for primates 85.8 Mya, and when comparing the placental mammals with the marsupial representative, an estimated divergence time of 95 Mya was computed. These results suggest that the primates are the oldest order of mammals analyzed in this study.

## Discussion

The objective of this study was to investigate if the HO-1 from the bottlenose dolphin exhibits modifications in its gene and protein structure as compared to that from terrestrial mammals. The *hmox1* of bottlenose dolphin and other taxa was analyzed using bioinformatics tools for the presence of binding sequences since, according to data from terrestrial mammals, HO-1 expression is controlled mostly at the transcription level (Choi and Alam, 1996). Previous studies report the presence of binding sequences for AP-1, AP-2, NFκB, HSF, and NF-E2 related factor-2, which upregulate *hmox1* (Lavrovsky et al., 1994; Inamdar et al., 1996). In addition, in this study, a binding sequence for STAT1:STAT2 in bottlenose dolphin *hmox1*, similar to the one reported for rat *hmox1*, was found (Lee et al., 2000). A binding sequence for STAT1:STAT2 was found in this study in all cetaceans species included and in pig *hmox1*; this motif was not found in the primate or mouse sequences. The transcription factor STAT acts as a signal transducer in the cytoplasm and transcription activators in the nucleus when cells encounter cytokines (Kisseleva et al., 2002). The phosphorylation of STAT1 and STAT2 results in heterodimers with interferon regulatory factor 9 and induces the expression of interferon-stimulated genes (Stark et al., 1998). Additionally, a binding sequence for CTCF, which controls the activation, repression and silencing of several genes (Chernukhin et al., 2007; Ohlsson et al., 2010), was found in *S. cerevisiae*, *Xenopus laevis* and the cetaceans analyzed in this study. In transgenic mice CTCF has been associated with the proximal E-box of the HO-1 gene (Kim et al., 2012).

HO is an integral enzyme of the smooth endoplasmic reticulum, with a transmembrane region at the C-terminus that functions as an anchor to said organelle (Kikuchi et al., 2005). This region, composed of a hydrophobic sequence, is conserved among all the mammalian species analyzed in this study. In cells exposed to hypoxia, HO-1 is found in the nuclear fraction, with a molecular weight of 28 kDa and truncated, with 52 amino acids missing at the C-terminal end (Lin et al., 2007). This form of the enzyme does not have catalytic activity but promotes the activation of AP-1, a transcription factor involved in cell proliferation (Lin et al., 2008) and antioxidant protection via regulation of glutamate-cysteine ligase, the rate-limiting enzyme in the production of glutathione (GSH). Higher concentration of GSH in the blood of marine mammals in comparison with terrestrial mammals has been reported (Vázquez-Medina et al., 2007). Since marine mammals are more frequently exposed to hypoxic events associated to I/R while diving, it is possible that a truncated HO-1 also serves as a transcription factor in these animals.

Secondary structures, such as α-helices, are essential for a protein’s function, and any change in the amino acid sequence can alter its nature. The HO-1 of bottlenose dolphin and human are 82.6% similar, leaving room for conformational changes, as suggested by the number of amino-acid substitutions. The arrangement and properties of amino-acids in a protein are key to the function and the understanding of its biological functions (Pal et al., 2006), since a single mutation can alter the function and structure, like the mutation in the glycine domain, Gly-139 or Gly-143, resulting in a loss of oxygenase activity (Liu et al., 2000). The lack of a glycine domain in the yeast, fruit flies (e.g., *Drosophila melanogaster*, *Ceratitis capitata*) and the wall cress (*Arabidopsis thaliana*) found in this study is in accordance with the report of Zhang et al. (2004), who found that Gly-143 is present, but Gly-139 is replaced by alanine, similar to the sequence of *A. thaliana* (Davis et al., 2001), suggesting that the heme pocket structure is different. Moreover, cysteine was found in several eukaryotic species analyzed in this study but primates and marine mammals. Inclusion of this amino-acid is considered to be a later addition to the genetic code (Trifonov, 2004), which appears to be under a firm evolutionary pressure. The lack of this residue in primate and marine mammal HO-1 may also be explained by the nature of the side chain of the cysteine, which can react in stress conditions that leads to increased oxidant levels (Shenton and Grant, 2003) such as the presence of pathogens; thus, a stress-inducible protein like HO-1 could benefit from the lack of an oxidant-sensitive amino-acid.

In this study, interactions of the heme group with residues at the active site were analyzed. Although, generally, the structure of bottlenose dolphin and human HO-1 shows few remarkable differences, a closer analysis revealed several changes in the amino-acid rotation. These changes in the plane rotation could be involved in the conformation of the active site’s amino-acids, thus, potentially affecting the enzyme’s activity. Even though the change of Ala to Thr (position 133) in the bottlenose dolphin HO-1 is a conserved substitution, the alanine side chain is non-reactive, so it is rarely involved in protein function; conversely, threonine, a quite common amino-acid in protein functional centers, has a reactive hydroxyl group (Barnes, 2007).

In this study, several residues in the bottlenose dolphin, as well as in other vertebrates, HO-1 (Figure 4) were found to have different rotation than in the human HO-1 (Supplementary Figure 4). The heme group has three propionates that help orient it and position the α meso carbon for hydroxylation; its interaction through a hydrogen bridge facilitates this hydroxylation. In the bottlenose dolphin HO-1, Lys-18, Lys-179, and Arg-183 were found to have a different rotation than that reported for the human HO-1. Since the heme group’s propionates can influence the protein’s electronic properties (Guallar and Olsen, 2006), this could indicate an alteration in the electronic properties of the bottlenose dolphin HO-1. The regioselectivity of HO-1 is due to the rotation of the heme group, and its configuration is dictated by the basic residues near the propionate site, Lys-18, Lys-22, Lys-179, and Arg-183 (Kikuchi et al., 2005). Similar to Lys-179 and Arg-183, Lys-18 and Lys-22 were observed to have a different rotation in the bottlenose dolphin than in the human HO-1; this could be taken to suggest a change in the regioselectivity of the bottlenose dolphin HO-1. Moreover, even though Lys-18 in the HO-1 from bottlenose dolphin has a closer interaction with the propionate than Lys-18 in the HO-1 from human, this must be interpreted with caution since the flexibility of the heme pocket has been demonstrated (Schuller et al., 1999; Wilks, 2002). To date, there is no evidence of a cetacean HO-1 crystal structure. The RMSD value calculated in this study for the bottlenose dolphin HO-1 showed a moderate value of alignment (0.55) with the human structure.

Evolutionary rates of proteins are not likely to be uniform, even in a group like mammals. Cetaceans are one of the mammalian lineages with more extensive adaptations to the new (aquatic) environmental conditions (Thewissen and Bajpai, 2001). Evidence of accelerated evolutionary rate was reported for cetacean myoglobin (Nery et al., 2013), suggesting a selective regime to withstand the aquatic demands. Although the fossil record of Cetacea provides a clear example of macroevolution, underlining the adaptive transition from terrestrial to aquatic lifestyle, in modern dolphins, like in the genus *Tursiops*, there is evidence of a rapid radiation with most of its divergence within the Pleistocene (2.58 Mya–117 kya). In this study, the phylogenetic tree of 24 species of mammals was constructed with HO-1 sequences; as expected, they were divided into distinct orders or superorders. Internal nodes represented the most recent common ancestors of at least two evolutionary lineages. A common ancestor of cetaceans and the common pig (67.4 Mya) was estimated, approximately 10 million years before cetacean transition from land to sea (Thewissen et al., 2007).

In this study, evidence of positive selection was detected by two methods. Although the sites with positive selection are within an α-helix configuration, it appears to have no change in the protein configuration. Moreover, when comparing cetaceans and primates, purifying selection was determined, indicating an evolutionary conservation of the coding sequence structure. Purifying (negative) selection favors an excess of synonymous vs. non-synonymous substitutions, preventing an amino acid residue’s change, thus sweeping away deleterious mutations (Monteiro et al., 2010). Both positive and negative selection are part of natural selection. In cetaceans, several hypoxia-tolerance-related genes were identified to be under positive selection (Tian et al., 2016). Molecular adaptations in the cytochrome b cetacean’s gene (McClellan et al., 2005), and evidence of positive selection in genes associated with the nervous system and deep diving (McGowen et al., 2012), have been reported. Combined, these results suggest that the cetacean genome is under selective pressure resulting in adaptive evolution to an aquatic lifestyle.

In marine mammals, molecular adaptations to oxidative stress and inflammation associated with hypoxic and ischemia/reperfusion stress have been suggested (Allen and Vázquez-Medina, 2019; Hindle, 2020). The adaptive evolution to withstand the so mentioned stresses is due to several genes, including hypoxia-inducible factor 1 (HIF-1) (Johnson et al., 2005; Bi et al., 2015), the glutathione system (Yim et al., 2014), peroxiredoxins (Zhou et al., 2018), and hemoglobin and myoglobin (Nery et al., 2013; Tian et al., 2016). Mammalian HO-1 is a well-regulated gene with several transcription factors that can bind to its proximal regulatory region. Even though this has been determined experimentally in the human HO-1 gene and several reports indicate the inducible nature of this gene, there is no experimental evidence in marine mammals such as the bottlenose dolphin. The results in this study show the utility of the public genome databases and software, providing an insight into the molecular structure and evolution of marine mammals’ HO-1, and the ability to generate novel hypotheses about the enzyme’s structure and function. Further studies evaluating the expression of HO-1 from marine mammals under diverse conditions are needed to assess its dynamic response and determine if the inflammatory-resolution response has changed with these species’ adaptations to an aquatic lifestyle. Additional studies could include further characterization, expression and activity of the heme oxygenase isoforms HO-2 and HO-3, which have been less studied, and there are no reports of these isoforms in marine mammals.

## Data Availability Statement

The original contributions presented in the study are included in the article/Supplementary Material, further inquiries can be directed to the corresponding author/s.

## Author Contributions

CR-R and TZ-S: study conception and design. CR-R: data collection. CR-R, RG-R, TZ-S, JV-M, LR-J, and OB-Q: analysis and interpretation of the results, draft manuscript, and preparation. All authors reviewed the results and approved the final version of the manuscript.

## Conflict of Interest

The authors declare that the research was conducted in the absence of any commercial or financial relationships that could be construed as a potential conflict of interest.

## Publisher’s Note

All claims expressed in this article are solely those of the authors and do not necessarily represent those of their affiliated organizations, or those of the publisher, the editors and the reviewers. Any product that may be evaluated in this article, or claim that may be made by its manufacturer, is not guaranteed or endorsed by the publisher.

## Footnotes

1
https://www.ncbi.nlm.nih.gov

2
https://www.encodeproject.org/

3
http://www.sbg.bio.ic.ac.uk/∽phyre2

4
http://pymol.sourceforge.net

5
https://www.ebi.ac.uk/Tools/msa/clustalo

6
http://www.timetree.org

7
http://www.bacteriamuseum.org/SWAAP/SwaapPage.htm
