# Supplementary material for: In silico Characterization of the Heme Oxygenase 1 From Bottlenose Dolphin (Tursiops truncatus): Evidence of Changes in the Active Site and Purifying Selection
Source: Front Physiol. 2021 Aug 12;12:711645. doi: 10.3389/fphys.2021.711645 (PMC8388933; doi:10.3389/fphys.2021.711645)
Supplement: Supplementary file 1 [file Data_Sheet_1.docx]

**Supplementary Figure 1.** Homology model of the bottlenose dolphin heme oxygenase-1 (HO-1). The transmembrane domain is shown in red. Helices 3_10_ are shown in brown, and the α-helices are shown in different colors. (A) The N and C terminal ends are shown; the heme group is found between the proximal (blue) and distal (light green) helices). (B) Model of the molecule rotated 180°.

**Supplementary Figure 2.** Homology modeling of the active site of bottlenose dolphin and human heme oxygenase-1 (HO-1). The residues closest to the heme group are indicated. Carbon atoms are shown in green for bottlenose dolphin and gray for human (except for the heme group, in which carbon atoms are shown in purple), nitrogen atoms in blue, and oxygen in red. (B) Model of the molecule rotated 180°.

**Supplementary Figure 3.** Multiple sequence analysis of mammalian heme oxygenase-1 (HO-1). In bold is histidine-25, the HO-1 ‘fingerprint’ is indicated, the glycine motif is enclosed in a box, and the transmembrane region for anchoring to the endoplasmic reticulum is underlined. Sites with positive and negative selection are indicated with (+) and (-), respectively. An asterisk * is for identical residues. α-helix, 3_10_ helix, and coil are indicated within the figure.

**Supplementary Figure 4.** Heme group interaction site of vertebrates’ heme oxygenase-1 (HO-1) alignment to human HO-1. Angle of rotation is shown in degrees. The carbon β is indicated in black. Carbon atoms are shown in gray for human, nitrogen atoms in blue, and oxygen in red. Mummichug (*Fundulus heteroclitus*) (purple), naked-mole rat (*Heterocephalus glaber*) (pink), deer mouse (*Peromyscus maniculatus bairdii*) (yellow), sperm whale (*Physeter catodon)* (green), rat (*Rattus rattus*) (cyan), California sea lion (*Zalophus californianus*) (blue), common frog (*Rana temporaria*) (wheat).
