# Supplementary figures and images for: In silico Characterization of the Heme Oxygenase 1 From Bottlenose Dolphin (Tursiops truncatus): Evidence of Changes in the Active Site and Purifying Selection
Source: Front Physiol. 2021 Aug 12;12:711645. doi: 10.3389/fphys.2021.711645 (PMC8388933; doi:10.3389/fphys.2021.711645)

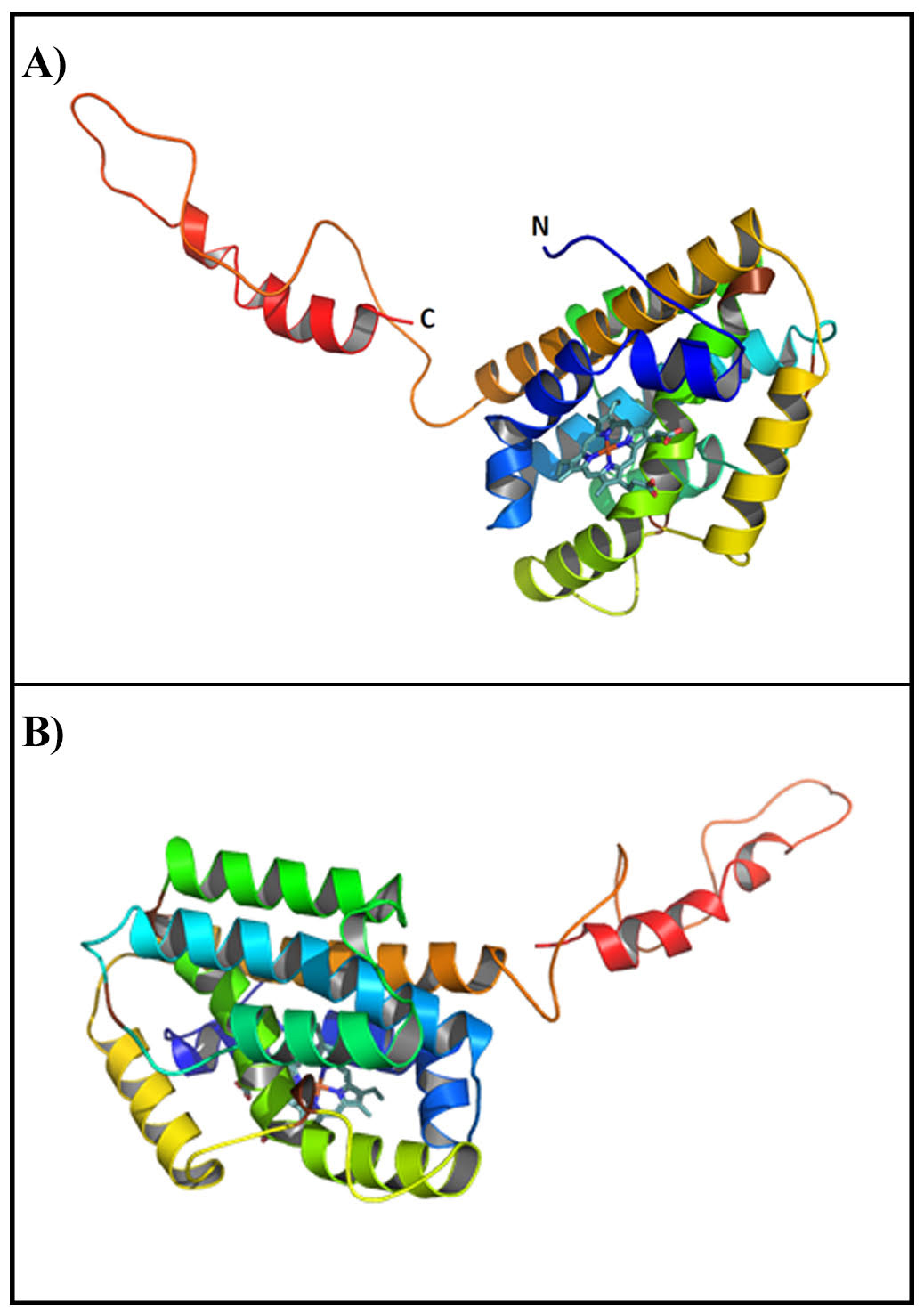

Supplement: Supplementary file 2 [file Image_1.JPEG]

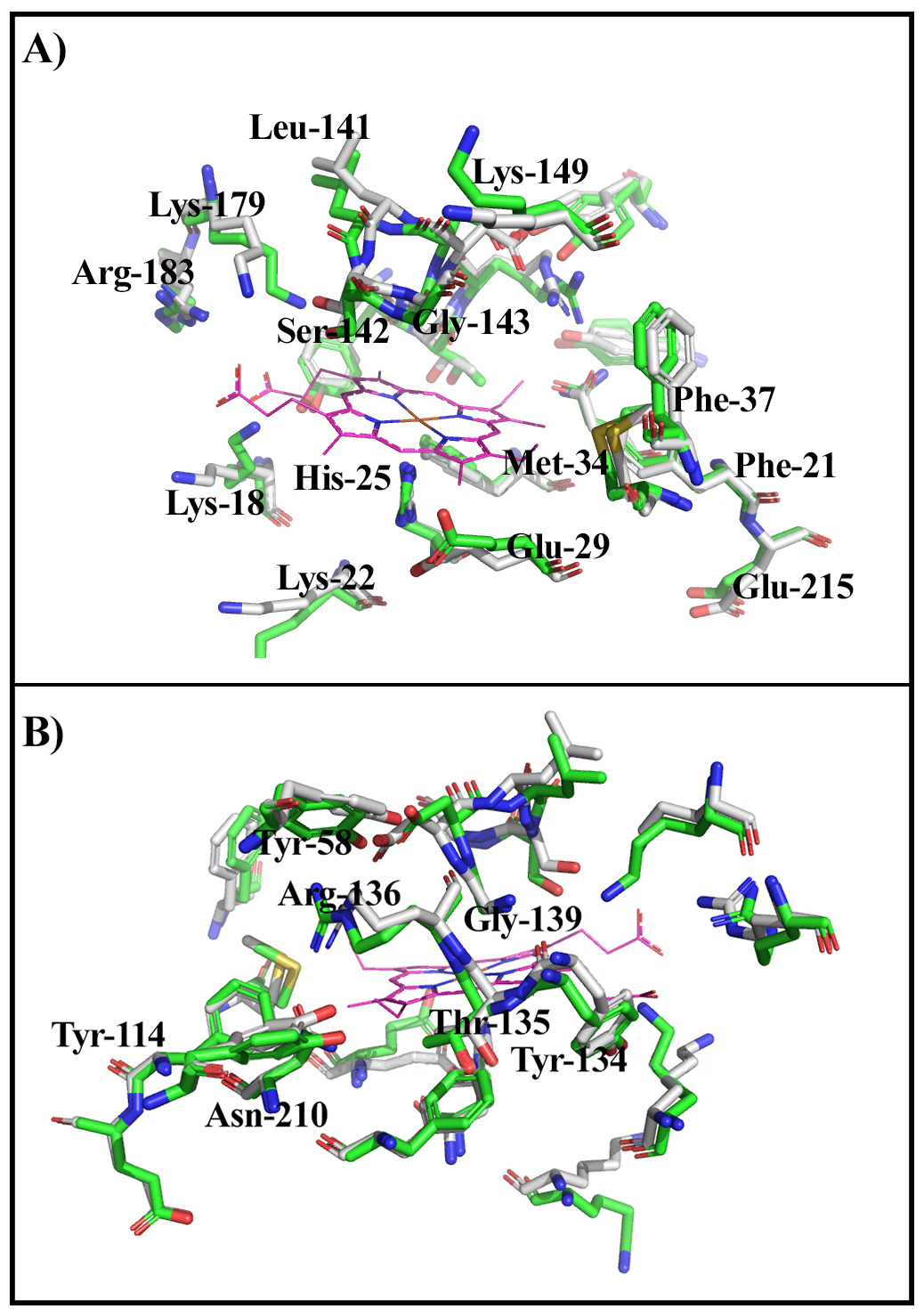

Supplement: Supplementary file 3 [file Image_2.JPEG]

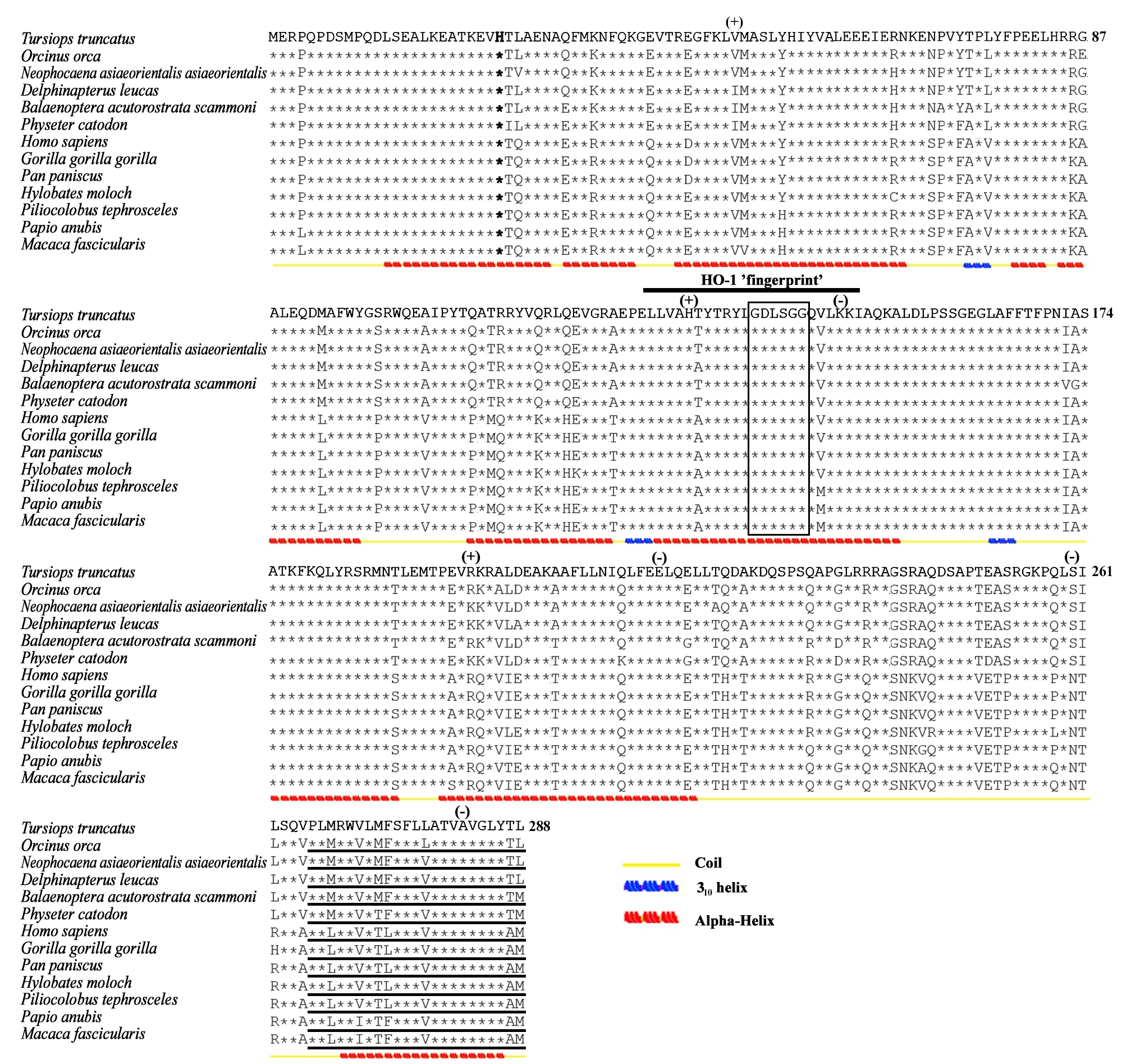

Supplement: Supplementary file 4 [file Image_3.JPEG]

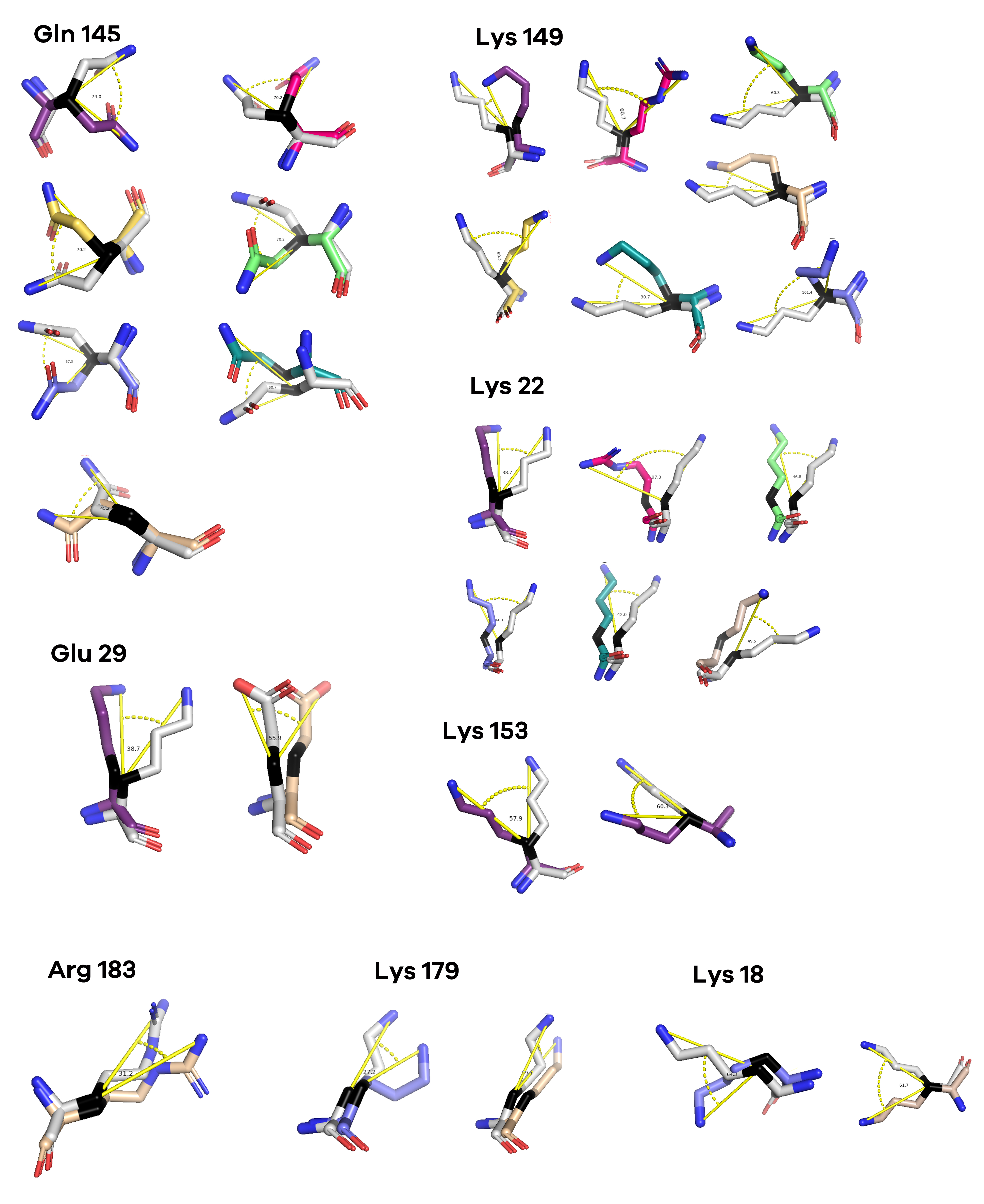

Supplement: Supplementary file 5 [file Image_4.JPEG]
